# Optically driving the radiative Auger transition

**DOI:** 10.1038/s41467-021-26875-8

**Published:** 2021-11-12

**Authors:** Clemens Spinnler, Liang Zhai, Giang N. Nguyen, Julian Ritzmann, Andreas D. Wieck, Arne Ludwig, Alisa Javadi, Doris E. Reiter, Paweł Machnikowski, Richard J. Warburton, Matthias C. Löbl

**Affiliations:** 1 Department of Physics, University of Basel, Klingelbergstrasse 82, 4056 Basel, Switzerland; 2 Lehrstuhl für Angewandte Festkörperphysik, Ruhr-Universität Bochum, 44780 Bochum, Germany; 3 Institut für Festkörpertheorie, Universität Münster, 48149 Münster, Germany; 4 Department of Theoretical Physics, Wrocław University of Science and Technology, 50-370 Wrocław, Poland

**Keywords:** Quantum dots, Quantum optics

## Abstract

In a radiative Auger process, optical decay leaves other carriers in excited states, resulting in weak red-shifted satellite peaks in the emission spectrum. The appearance of radiative Auger in the emission directly leads to the question if the process can be inverted: simultaneous photon absorption and electronic demotion. However, excitation of the radiative Auger transition has not been shown, neither on atoms nor on solid-state quantum emitters. Here, we demonstrate the optical driving of the radiative Auger transition, linking few-body Coulomb interactions and quantum optics. We perform our experiments on a trion in a semiconductor quantum dot, where the radiative Auger and the fundamental transition form a Λ-system. On driving both transitions simultaneously, we observe a reduction of the fluorescence signal by up to 70%. Our results suggest the possibility of turning resonance fluorescence on and off using radiative Auger as well as THz spectroscopy with optics close to the visible regime.

## Abstract (web summary)

Radiative Auger is a process that leads to a red-shift of the optical emission of an atom or a charged solid-state quantum emitter. Here, the authors realize the inverse process by optically driving the radiative Auger transition of a short-lived electronic state in a semiconductor quantum dot.

## Introduction

Non-radiative Auger processes have been observed in both atoms^1^ and solid-state quantum emitters^2,3^. They play an important role in determining the efficiency of semiconductor light-emitting diodes and lasers^4^. In the non-radiative Auger process, one electron reduces its energy by transferring it to a second electron that is promoted to a high-energy state. In the radiative Auger process (shake-up), in contrast, one electron makes an optical decay, creating a photon. Part of the photon energy is transferred to a second electron such that the radiative Auger emission is red-shifted with respect to the main emission line. Both radiative and non-radiative Auger processes arise as a consequence of the Coulomb interactions between electrons in close proximity^5–7^. Non-radiative Auger is a purely Coulomb scattering process. In contrast, radiative Auger involves both Coulomb scattering and electron-photon interactions. It can either be viewed as a higher-order scattering process or interpreted in terms of Coulomb-induced admixtures of higher single-particle states to the multi-electron wave function^7,8^. What appears to be an optical relaxation of one electron in the single-particle picture involves, in fact, a sudden change of the many-particle configuration.

Radiative Auger emission has been observed over a large spectral range: in the X-ray emission of atoms^9^; close to visible frequencies on donors in semiconductors^10^ and quantum emitters^11,12^; and at infrared frequencies as shake-up lines in two-dimensional systems^13–17^. Furthermore, radiative Auger connects carrier dynamics to the quantum optical properties of the emitted photons^11^, making it a powerful probe of multi-particle systems. Driving the fundamental transition between the electron ground state and an optically excited state is an important technique in quantum optics^18,19^. In contrast, driving the radiative Auger transition has not been achieved, neither on atoms nor on solid-state systems. Success here would significantly increase the number of quantum optics techniques that can be employed.

**Fig. 1:**
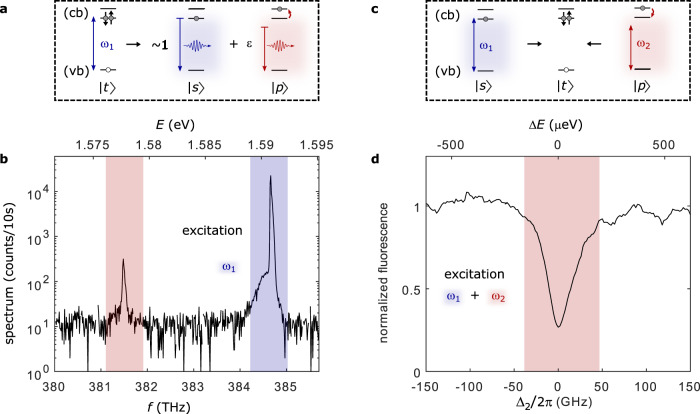
Radiative Auger emission and excitation of the radiative Auger transition. **a** Schematic illustration of the fundamental transition and the radiative Auger process. The trion state $\left|t\right\rangle$ optically decays by recombination of one electron in the conduction band (cb) with a hole in the valence band (vb). The second electron either stays in its ground state $\left|s\right\rangle$ (fundamental transition), or is left in a higher shell $\left|p\right\rangle$ (radiative Auger). The radiative Auger photon is red-shifted from the fundamental transition by the energy transferred to the Auger electron. **b** Emission spectrum from a negatively charged quantum dot upon optical excitation at the fundamental transition. In addition to the fundamental transition (highlighted in blue), there is a red-shifted satellite line (highlighted in red). This emission arises from the radiative Auger process, where the trion state $\left|t\right\rangle$ decays to the excited electron state $\left|p\right\rangle$. **c** Two possible absorption channels in the presence of one confined conduction band electron. When the electron is in the ground state $\left|s\right\rangle$, a laser resonant with the fundamental transition (blue, frequency *ω*_1_, Rabi frequency Ω_1_) excites a valence band electron and brings the system to the trion state, $\left|t\right\rangle$. When the conduction band electron is in an excited state $\left|p\right\rangle$, a red-shifted laser (frequency *ω*_2_, Rabi frequency Ω_2_) can excite the system to the same trion state $\left|t\right\rangle$. In this inverted radiative Auger process, the missing energy is provided by the excited electron. **d** Resonance fluorescence from the fundamental transition in the presence of a strong second laser. When the second laser (*ω*_2_) is on resonance with the radiative Auger transition (Δ_2_ = 0), the resonance fluorescence intensity is strongly reduced.

We demonstrate driving the radiative Auger transition on an epitaxial GaAs quantum dot embedded in AlGaAs^20,21^. The quantum dot forms a potential minimum and confines charge carriers, resulting in discrete energy levels like in an atom. Without optical illumination, a single electron is trapped in the conduction band of the quantum dot and occupies the lowest possible shell (the *s*-shell, $\left|s\right\rangle$). Upon resonant excitation of the fundamental transition, a second electron is promoted from the filled valence band to the conduction band and a negative trion *X*^1−^ ($\left|t\right\rangle$) is formed. This trion consists of two electrons in the conduction band and one electron-vacancy (hole) in the valence band. Figure 1a shows the possible optical decay paths: in the fundamental transition, one electron decays, removing the valence band hole while the other electron remains in the conduction band ground state $\left|s\right\rangle$; in the radiative Auger process, the remaining electron is left in an excited state $|p\rangle$. The emitted photon is red-shifted by the energy separation between $|p\rangle$ and $\left|s\right\rangle$^5,11^. Figure 1b shows a typical emission spectrum from the trion decay. This spectrum is measured on resonantly driving the fundamental transition $\left|s\right\rangle$–$\left|t\right\rangle$ at 384.7 THz (1.591 eV) with a narrow-bandwidth laser^11^. Red-shifted by 3.2 THz (13.2 meV) from the fundamental transition, there is a weak satellite line that arises from the radiative Auger process.

Photons at the radiative Auger frequency have insufficient energy to excite the fundamental transition $\left|s\right\rangle$–$\left|t\right\rangle$. Figure 1c shows how the trion state $\left|t\right\rangle$ still can be excited with a laser at the Auger transition. The missing energy is provided by the electron, which initially occupies the excited state $|p\rangle$. However, driving the radiative Auger transition is experimentally challenging for two main reasons: first, there is a fast non-radiative relaxation from the excited single-electron state $|p\rangle$ back to $\left|s\right\rangle$^11,22^, and the state $|p\rangle$ is not occupied at thermal equilibrium. Second, the dipole moment of the radiative Auger transition is small. Therefore, it is difficult to achieve high Rabi frequencies on driving the transition, plus the radiative Auger emission is very weak and hard to distinguish from the back-reflected laser light.

## Results

We perform a two-laser experiment revealing optical driving of the radiative Auger transition. The fundamental transition $|s\rangle$–$\left|t\right\rangle$ (at ~ 1.591 eV) is driven with one laser (labelled by *ω*_1_) while the radiative Auger transition (at ~ 1.578 eV) is simultaneously driven with a second laser (labelled by *ω*_2_). This Λ-configuration has the following advantages: First, on driving $\left|s\right\rangle$–$|t\rangle$ with *ω*_1_, there is a small chance of initializing the system in state $|p\rangle$ via the radiative Auger emission. Additionally, driving the $|p\rangle$–$\left|t\right\rangle$-transition with *ω*_2_, while transferring population to $\left|t\right\rangle$ with *ω*_1_ also leads to a finite occupation of $|p\rangle$. Second, the small dipole matrix element of the radiative Auger transition is compensated by using high power for *ω*_2_. The high power causes a high laser background when detecting the fluorescence from the radiative Auger transition. Instead, we tune the second laser over the Auger transition while measuring just the fluorescence originating from the fundamental transition $\left|s\right\rangle$–$\left|t\right\rangle$. Figure 1d shows the result of this two-laser experiment. We observe a strong reduction in fluorescence on addressing the transition $|p\rangle$–$\left|t\right\rangle$ which is characteristic of two-colour excitation of a Λ-configuration. Our approach has a conceptual similarity to the driving of weak phonon sidebands of mechanical resonators resulting in optomechanically induced transparency^23,24^.

### Autler–Townes splitting in single-laser experiments

**Fig. 2:**
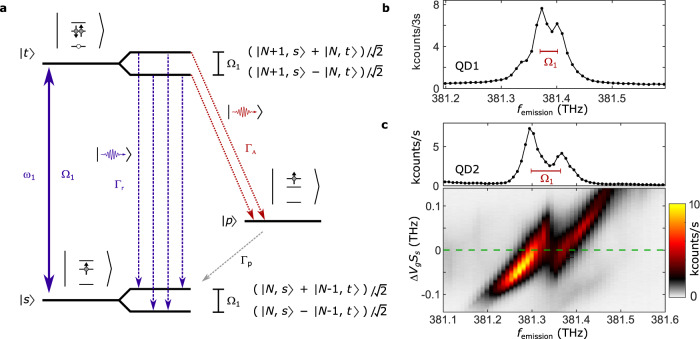
Autler–Townes splitting in the radiative Auger emission. **a** Level scheme under strong resonant driving of the fundamental transition ($\left|s\right\rangle$−$\left|t\right\rangle$). The energy levels of the transition are split into dressed states. The splitting between the dressed states is given by the Rabi frequency, Ω_1_. In the radiative Auger emission (red arrows), the dressed-state splitting creates two decay paths leading to an Autler–Townes splitting. **b** Radiative Auger emission from the main quantum dot (QD1) on driving the transition $\left|s\right\rangle$−$\left|t\right\rangle$ with *ω*_1_. The Rabi frequency of Ω_1_ = 2*π* × 31.9 GHz (red bar) is determined from a power saturation curve. The measured Autler–Townes splitting in the emission matches the Rabi frequency. **c** Emission spectrum from a second quantum dot (QD2), measured for a set of different detunings (Δ_1_ = Δ*V*_*g*_ ⋅ *S*_*s*_) between fundamental transition and laser frequency. The upper part of the plot is a line cut along the dashed green line at zero detuning (Δ_1_ = 0). In this case, the measured Autler–Townes splitting agrees with the independently determined Rabi frequency (Ω_1_ = 2*π* × 67.7 GHz).

We consider initially the situation where the fundamental transition ($\left|s\right\rangle$–$\left|t\right\rangle$) is strongly driven by a single laser. If radiative Auger and fundamental transition form a Λ-system, one would expect an Autler–Townes splitting in the radiative Auger emission. Figure 2a shows the corresponding level scheme including the dressed states $\frac{1}{\sqrt{2}}(\left|N+1,s\right\rangle \pm \left|N,t\right\rangle )$ and $\frac{1}{\sqrt{2}}(\left|N,s\right\rangle \pm \left|N-1,t\right\rangle )$, where *N* is the photon number. The dressed-state splitting leads to the Mollow triplet in the resonance fluorescence^19,25,26^. For a decay into a third level, the Autler–Townes splitting^27,28^ in the emission is expected to be Ω_1_. Figure 2b shows the radiative Auger emission of one quantum dot (QD1). In this measurement, the laser is on resonance with the fundamental transition. The Rabi frequency (Ω_1_ = 2*π* × 31.9 GHz, red bar in Fig. 2b) is estimated independently by measuring the fluorescence intensity as a function of laser power (Supplementary Fig. 3b). We observe an Autler–Townes splitting that agrees well with this Rabi frequency. For this quantum dot, we also observe an additional weak emission appearing on the low energy side of the spectrum when using high Rabi frequencies (Fig. 2b and Supplementary Fig. 4). We speculate that this emission is connected to optical coupling between $|p\rangle$ and an excited trion state, $\left|{t}^{* }\right\rangle$. Figure 2c shows radiative Auger emission from a second quantum dot (QD2). For this quantum dot, we measure the radiative Auger emission as a function of detuning between the quantum dot transition and the laser (see Supplementary Fig. 4 for the corresponding measurement on QD1). On applying a gate voltage Δ*V*_*g*_, the quantum dot transition $\left|s\right\rangle$–$\left|t\right\rangle$ is detuned from the fixed laser by Δ_1_ = Δ*V*_*g*_ ⋅ *S*_*s*_ via the quantum-confined Stark shift. *S*_*s*_ parameterizes the Stark shift of the fundamental transition. At zero detuning, the observed Autler–Townes splitting again agrees with the Rabi frequency obtained from a power saturation curve (Ω_1_ = 2*π* × 67.7 GHz).

### Two-laser experiments

**Fig. 3:**
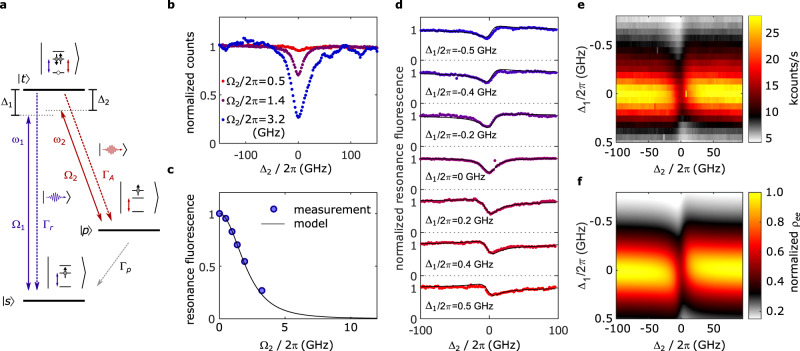
Optically driving the radiative Auger transition. **a** The level scheme where one laser (*ω*_1_) with Rabi frequency Ω_1_ drives the fundamental transition ($\left|s\right\rangle$–$\left|t\right\rangle$) while a second laser (*ω*_2_) drives the radiative Auger transition ($\left|p\right\rangle$–$\left|t\right\rangle$) with Rabi frequency Ω_2_. **b** Resonance fluorescence (Ω_1_ = 2*π* × 0.08 GHz) as a function of detuning Δ_2_ (detuning of *ω*_2_). At low values of Ω_2_, the resonance fluorescence intensity is almost constant for different values of Δ_2_. For the highest value of Ω_2_, the resonance fluorescence drops by up to ~ 70% on bringing *ω*_2_ into resonance with the radiative Auger transition. The strong fluorescence dip at a particular frequency is a characteristic feature of a Λ-system driven with two lasers that are detuned in frequency by the ground state splitting. **c** Resonance fluorescence at Δ_2_ = 0 as a function of Ω_2_. The resonance fluorescence intensity (blue dots) drops with increasing Ω_2_, fitting well to the theoretical model (black line). **d** Fluorescence intensity as a function of detuning Δ_2_. The Rabi frequencies are Ω_1_ = 2*π* × 0.27 GHz, Ω_2_ = 2*π* × 2.1 GHz. The same measurement is repeated for a series of fixed detunings Δ_1_ (detuning of *ω*_1_ from the fundamental transition). Detuning *ω*_1_ leads to an asymmetric fluorescence dip. This asymmetry is well captured by our quantum optics simulations (black lines) based on the level scheme shown in (**a**). **e** Fluorescence intensity as a function of laser detunings Δ_1_, Δ_2_. **f** Simulation of the fluorescence intensity as a function of the laser detunings.

We now consider the experiments with the second laser (labelled as *ω*_2_) at the radiative Auger transition. Figure 3a shows the corresponding level scheme. We set *ω*_1_ to a modest Rabi frequency (Ω_1_ = 2*π* × 0.08 GHz) compared to the decay rate of the trion (Γ_*r*_ = 2*π* × 0.50 GHz). The frequency of the radiative Auger transition is estimated from the trion emission spectrum (Fig. 1b). We sweep the frequency *ω*_2_ and simultaneously monitor the resonance fluorescence intensity from the fundamental transition. Figure 3b shows this measurement for different powers of the laser on the Auger transition. On increasing the power of *ω*_2_ to several orders of magnitude higher than the power of *ω*_1_, there is a pronounced dip in the fluorescence intensity. This intensity dip appears precisely when the laser frequency *ω*_2_ matches the radiative Auger transition ($|p\rangle$–$\left|t\right\rangle$) and is characteristic for a Λ-system that is driven with two lasers. We estimate the Rabi frequency Ω_2_ driving $|p\rangle$–$\left|t\right\rangle$ by simulating the resonance fluorescence intensity as a function of Δ_2_ (see Supplementary Note 1 for the quantum optics simulation). In this simulation, we keep the decay rate from $|p\rangle$ to $\left|s\right\rangle$ (Γ_*p*_ ~ 2*π* × 9.3 GHz) fixed to the value that we determine from independent auto- and cross-correlation measurements^11^ (Supplementary Fig. 2d). The value for Ω_2_ can then be determined by a corresponding fit to the two-laser experiment. Additionally, we fit a constant pure dephasing, *γ*_*p*_, for the state $|p\rangle$ which leads to an additional broadening of the fluorescence dip. We estimate *γ*_*p*_ ~ 2*π* × 8.8 GHz from the fit and a Rabi frequency of Ω_2_ = 2*π* × 3.2 GHz (*ω*_2_) for the strongest fluorescence dip. Note that additional excitation-induced dephasing via phonons is expected to be weak for such Rabi frequencies^29,30^.

In Fig. 3c, we plot the minimum of the resonance fluorescence dip as a function of Ω_2_. The Λ-system model with two driving lasers fits this data set well. For the highest value of Ω_2_, we achieve a reduction of the resonance fluorescence intensity by up to 70%. The intensity reduction is limited by the power that we can reach in our optical setup. The measurement shows that resonance fluorescence can be switched on and off by using the radiative Auger transition. In our system, part of the fluorescence dip is due to the reduction of the overall absorption via the formation of a dark state. This effect is related to electromagnetically induced transparency (EIT)^31^ and coherent population trapping (CPT)^32,33^. An additional reduction of the signal comes from the fact that there is a fast decay rate Γ_*p*_ from state $|p\rangle$ to $\left|s\right\rangle$. Thus, after the laser-induced transition from state $\left|t\right\rangle$ to $|p\rangle$, the system quickly decays to the ground state $\left|s\right\rangle$. This de-excitation channel reduces the population of the trion state and, therefore, the fluorescence intensity. We can distinguish the contributions of the two mechanisms by our quantum optics simulation. The density matrix element *ρ*_*tt*_ (occupation of state $\left|t\right\rangle$) is proportional to the overall fluorescence intensity. The term Im(*ρ*_*st*_) (coherence between the states $\left|s\right\rangle$ and $\left|t\right\rangle$) is proportional to the absorption and reflects the coherent part of the intensity reduction. The contribution of both mechanisms is comparable for the parameter regime in which we operate (Supplementary Fig. 1b).

The measurements so far were performed with *ω*_1_ on resonance (Δ_1_ = 0). We repeat the two-laser experiments while detuning *ω*_1_ from the fundamental transition. Figure 3d shows the fluorescence intensity for positive, zero, and negative detuning Δ_1_. For non-zero detuning, the fluorescence dip is asymmetric as a function of Δ_2_. The asymmetry is an important result as it cannot be explained by a rate equation description, but depends on the quantum coherence in the master equation model (Supplementary Note 1). The full dependence of the resonance fluorescence intensity as a function of Δ_1_ and Δ_2_ is plotted in Fig. 3e. This data set fits well to the corresponding quantum optics simulation in Fig. 3f using the parameters from the previous measurements.

## Discussion

Upon the optical transition of a carrier, radiative Auger leaves other carriers in an excited orbital state, and the emitted photon is red-shifted. We show here that this process can be inverted: radiative Auger exists in absorption and the corresponding transition can be optically driven. In both emission and absorption, the process has conceptual similarities to phonon scattering. For radiative Auger emission, the electronic configuration is left in an excited state, for the phonon sideband, the lattice configuration^34,35^. We demonstrate that the resonance fluorescence can be strongly reduced by addressing the radiative Auger transition: a modulated laser on the radiative Auger transition could be used for fast optical gating of the emitter’s absorption. As an outlook, we suggest that an effective coupling between orbital states, split by frequencies in the THz band, can be created by two lasers at optical frequencies. The idea here is to establish a Raman-like process: the lasers are equally detuned from their resonances, and an exciton is not created. This scheme facilitates control of the orbital degree of freedom with techniques that have been developed for manipulating spin-states^33,36^. Further quantum optics experiments with radiative Auger photons are conceivable: by using a two-colour Raman-scheme^37^, it might be possible to create deterministically highly excited shake-up states that are also subject of recent theoretical investigations^38^. Adding a third laser with a THz-frequency at the transition $\left|s\right\rangle$–$|p\rangle$^22^ might even allow close-contour driving schemes^39^. In analogy to experiments on spins^40^, the radiative Auger process could lead to an entanglement between the frequency of the emitted photon and the orbital state of the Auger electron.

## Methods

For all our measurements, the quantum dot sample is kept in a liquid helium bath cryostat at 4.2 K. The quantum dots used in this work are GaAs quantum dots in AlGaAs grown by molecular beam epitaxy. Their decay rates Γ_*r*_ (typically in the range 2*π* × (0.5 − 0.6) GHz) were determined by lifetime measurements using pulsed resonant excitation^21^. The decay rate of the radiative Auger transition, Γ_*A*_ ~ Γ_*r*_/100, is estimated by comparing its emission intensity to the fundamental transition. QD1 is identical to the second quantum dot in Ref. ^21^. The quantum dots presented in this work have a stronger radiative Auger emission compared to other III-V quantum dots^11^ indicating a stronger dipole moment of the radiative Auger transition. We use radiative Auger lines where the final state of the Auger electron, $|p\rangle$, is a quantum dot *p*-shell. In particular, we investigate the transition associated with the lower *p*-shell (*p*_+_) for QD1 and the higher *p*-shell (*p*_−_) for QD2. We can assign further emission lines to the corresponding higher electronic shells by measuring the magnetic field dispersion of the emission spectrum^11^ (Supplementary Fig. 3a).

To excite the quantum dots, we use a tunable diode laser with a narrow bandwidth ( ~ 100 kHz) far below the quantum dot linewidth. Resonant excitation is not necessary to observe the radiative Auger emission: above-band excitation is also effective^7,12^. It is also possible to observe radiative Auger on systems that suffer from much more charge noise than ours^12^. However, resonant excitation has the advantage that no continuum states are excited making it easier to identify all emission lines, and low charge noise makes resonant excitation a lot easier to perform. For this work, resonant excitation is crucial to optically address a single radiative Auger transition. To suppress the reflected excitation laser, we use a cross-polarization scheme^41^.

To determine the relaxation rate Γ_*p*_ ( ~2*π* × 9.3 GHz) from $|p\rangle$ to $\left|s\right\rangle$, we make use of a technique developed in Ref. ^11^: on driving $\left|s\right\rangle$–$\left|t\right\rangle$ (Ω_2_ = 0), we measure an auto-correlation of the resonance fluorescence from the fundamental transition and compare it to the cross-correlation between resonance fluorescence from the fundamental transition and radiative Auger emission. The corresponding measurement setups are shown in Supplementary Fig. 5a, b. To resolve the auto- and cross-correlations with high time resolution, we use two superconducting nanowire single-photon detectors (SingleQuantum) with a timing jitter below 20 ps (FWHM) in combination with correlation hardware (Swabian Instruments).

Compared to the auto-correlation, the cross-correlation has a small time offset when a radiative Auger photon is followed by a photon from the fundamental transition (Supplementary Figs. 2d, e). This time scale corresponds to the relaxation time, *τ*_*p*_ = 1/Γ_*p*_, describing the relaxation from $|p\rangle$ to $\left|s\right\rangle$. The relaxation time appears in the cross-correlation: when a radiative Auger event is detected by the first detector, there is an additional waiting time of *τ*_*p*_ before the excited Auger electron relaxes to the ground state and the system can be optically re-excited. Therefore, it takes longer before a second photon is detected. The additional waiting time is only present for the cross-correlation. For the auto-correlation, the system decays directly to the ground state $\left|s\right\rangle$ and there is no additional waiting time.

Finally, we also measure the auto-correlation of the radiative Auger emission (see Supplementary Fig. 5c for the setup). The measurement is shown in Supplementary Fig. 5e. We observe a pronounced anti-bunching at zero delays proving the single-photon nature of the radiative Auger photons. Going beyond the results in Ref. ^11^, we observe the Rabi oscillation from strongly driving the transition $\left|s\right\rangle$–$\left|t\right\rangle$ in the photon-statistics of the radiative Auger photons from the transition $|p\rangle$–$\left|t\right\rangle$.

## Supplementary information

supplementary information

## Data Availability

The data that supports this work is available from the corresponding author upon reasonable request.

## Code availability

The code that has been used for this work is available from the corresponding author upon reasonable request.
