# Supplementary material for: Optically driving the radiative Auger transition
Source: Nat Commun. 2021 Nov 12;12:6575. doi: 10.1038/s41467-021-26875-8 (PMC8590044; doi:10.1038/s41467-021-26875-8)
Supplement: Supplementary file 1 — supplementary information [file 41467_2021_26875_MOESM1_ESM.pdf]

Supplementary Information – Optically driving the radiative Auger transition

Clemens Spinnler[1,*], Liang Zhai[1,*], Giang N. Nguyen[1], Julian Ritzmann[2], Andreas D. Wieck[2], Arne Ludwig[2], Alisa Javadi[1], Doris E. Reiter[3], Paweł Machnikowski[4], Richard J. Warburton[1], and Matthias C. Löbl[1,*,†]

[1] *Department of Physics, University of Basel, Klingelbergstrasse 82, 4056 Basel, Switzerland*
[2] *Lehrstuhl für Angewandte Festkörperphysik, Ruhr-Universität Bochum, 44780 Bochum, Germany*
[3] *Institut für Festkörpertheorie, Universität Münster, 48149 Münster, Germany*
[4] *Department of Theoretical Physics, Wrocław University of Science and Technology, 50-370 Wrocław, Poland*

[*] *These authors contributed equally to this work.*
[†] *Correspondence should be addressed to: matthias.loebl@unibas.ch*

SUPPLEMENTARY NOTE 1: MODELLING THE Λ-SYSTEM

The level scheme to describe the two-laser experiments is shown in Supplementary Fig. 1. It consists of the electron ground state $|s\rangle$, an excited electron state $|p\rangle$, and the trion state $|t\rangle$. The laser driving the fundamental transition is labelled as ω_1 and the laser driving the radiative Auger transition is labelled as ω_2. The corresponding Rabi frequencies are given by Ω_1, Ω_2 and the detunings of the lasers from the corresponding transition are Δ_1, Δ_2. The spontaneous decay rates are the decay rate via the fundamental transition (Γ_r), the decay rate via radiative Auger (Γ_A), and the p-to-s decay rate (Γ_p). We simulate the system with a standard quantum optics approach. Making the dipole and the rotating-wave approximations, the Hamiltonian of the system is given by[1,2]:

$$\hat{H} = \frac{\hbar}{2}[2(\Delta_2 - \Delta_1)|p\rangle\langle p| - 2\Delta_1 |t\rangle\langle t| + \Omega_1 |t\rangle\langle s| + \Omega_2 |t\rangle\langle p| + \Omega_1 |s\rangle\langle t| + \Omega_2 |p\rangle\langle t|]. \quad (1)$$

The Hamiltonian describes the coherent evolution of the system. The incoherent decay paths are taken into account by the Lindblad collapse operators for the spontaneous emission from the fundamental transition ($L_1 = \sqrt{\Gamma_r}\,|s\rangle\langle t|$), the spontaneous radiative Auger emission ($L_2 = \sqrt{\Gamma_A}\,|p\rangle\langle t|$), the p-to-s relaxation ($L_3 = \sqrt{\Gamma_p}\,|s\rangle\langle p|$), and the p-shell dephasing ($L_4 = \sqrt{\gamma_p}\,|p\rangle\langle p|$). The dynamics of the system are described by the following master equation:

$$i\hbar\frac{d\rho}{dt} = [\hat{H}, \rho] + i\hbar\sum_i \left(L_i \rho L_i^\dagger - \frac{1}{2}\{L_i^\dagger L_i, \rho\} \right), \quad (2)$$

where ρ is the density matrix. Using this equation, we determine the steady state of the system ($\frac{d\rho}{dt} = 0$). The steady state occupation of the trion state is used for simulating the experiments as it is proportional to the fluorescence intensity.

This simulation fits well to our experimental results in Fig. 3 of the main text. We also use it to estimate the Rabi frequency Ω_2 and the dephasing γ_p: when Δ_2 is close to zero, the resonance fluorescence depends on Ω_2.

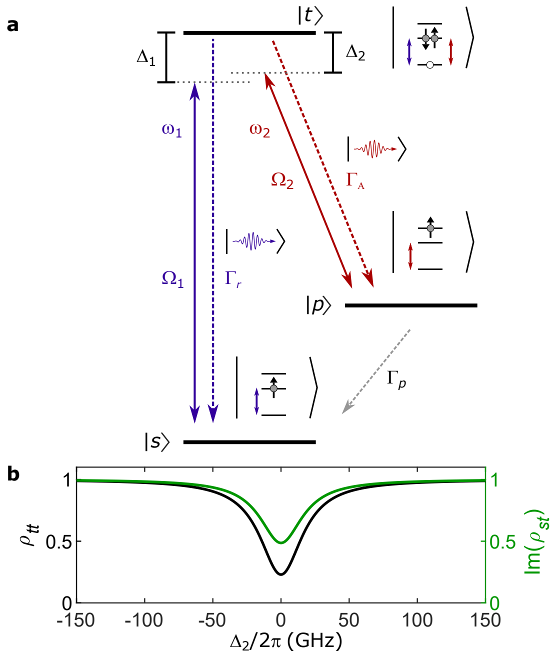

Supplementary Fig. 1. **Level scheme and quantum optics simulation. (a)** Fundamental transition and radiative Auger transition form a Λ-system where both transitions can be driven by two independent lasers. The Rabi-frequency of the laser on the fundamental transition (ω_1) is given by Ω_1, the Rabi frequency of the laser on the radiative Auger transition (ω_2) is given by Ω_2. The corresponding laser detunings are Δ_1 and Δ_2, the corresponding spontaneous decay rates from the trion state $|t\rangle$ are Γ_r (fundamental transition), Γ_A (radiative Auger). The parameter Γ_p is the relaxation rate from the electron excited state $|p\rangle$ to the electron ground state $|s\rangle$. **(b)** Comparison of the density matrix elements ρ_{tt} and $\mathrm{Im}(\rho_{st})$ as a function of Δ_2. The parameters are identical to those used to describe the deepest fluorescence dip shown in Fig. 3(b) of the main text.

Due to the small dipole moment of the radiative Auger transition, strong laser powers are required to achieve high values of Ω_2. For the strongest laser power of ω_2 (increasing the power of ω_1 by a factor of $\sim 8 \times 10^3$), we estimate $\Omega_2 = 2\pi \times 3.2$ GHz from the simulation. Alternatively, one could estimate the ratio of the corre-

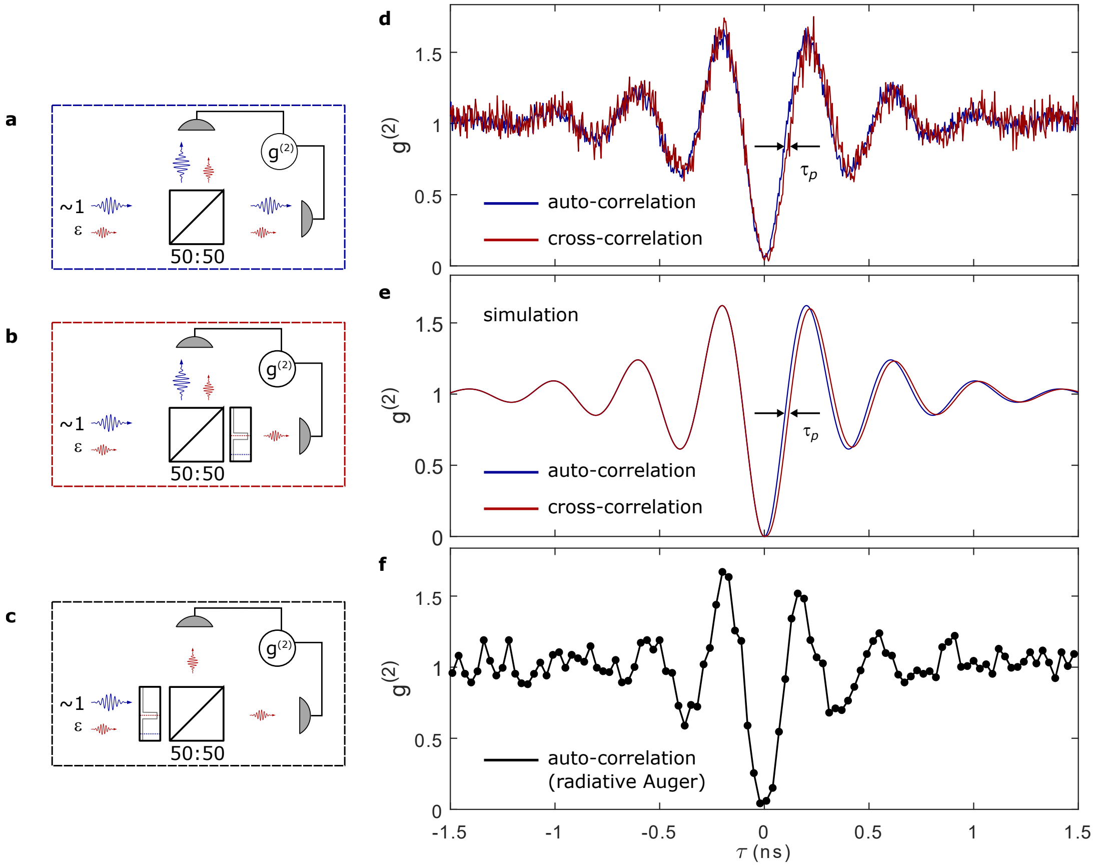

Supplementary Fig. 2. **Time-resolved correlation measurements.** **(a)** Schematic measurement setup for the auto-correlation of resonance fluorescence from the fundamental transition. **(b)** Schematic setup for the cross-correlation between resonance fluorescence and radiative Auger emission. **(c)** Schematic setup for the auto-correlation of the radiative Auger emission. **(d)** Comparison between the auto-correlation of the resonance fluorescence (blue) and the cross-correlation between resonance fluorescence and radiative Auger emission (red, data from QD1). Both correlation-measurements ($g^{(2)}$) are performed with a single laser on the fundamental transition and show Rabi oscillations due to the strong driving (Ω_1). The cross-correlation has a small offset given by the $|p\rangle$-to-$|s\rangle$ relaxation time ($\tau_p = 1/\Gamma_p = 17$ ps). This offset measures the finite time for which the Auger electron remains in the excited state after a radiative Auger process has occurred[3]. The origin of the relaxation Γ_p is probably a phonon-assisted decay[4] but further investigations are needed. **(e)** Simulation of the measurements shown in (d). **(f)** Auto-correlation of the radiative Auger emission. Since the radiative Auger emission is relatively weak (count rates: 630 Hz on the first, 530 Hz on the second detector), a long integration time ($\sim$ 50 h) is needed to resolve the Rabi oscillations in this measurement. The excitation power and Rabi frequency are slightly different with respect to the auto- and cross-correlation shown in (d).

sponding dipole moments by using the intensity ratio between resonance fluorescence and radiative Auger emission ($\sim$ 50 : 1). Ω_2 could then be obtained by using this estimation together with the power saturation curve of the resonance fluorescence. We find that this method underestimates Ω_2 compared to the simulation. Since effects such as chromatic aberration make this second approach more prone to systematic errors, we always use the two-laser experiment and the corresponding simulation to determine Ω_2. The dephasing term γ_p is also estimated by simulating the two-laser experiment. We find that it mainly affects the width of the fluorescence dip. As explained in the main text, other parameters (Γ_r, Γ_p, Ω_1) are determined from independent measurements and kept fixed in the simulation.

There are two mechanisms that contribute to the fluorescence reduction when driving the Auger transition with ω_2: a coherent part related to EIT/CPT and dark state formation[1], an incoherent part due to a fast de-excitation channel from $|t\rangle$ to $|p\rangle$ via radiative Auger and from $|p\rangle$ to $|s\rangle$ by two-phonon emission[4]. The incoherent decay path is irrelevant in systems where the ground state lifetime is long[1]. To distinguish these two mechanisms we compare the density matrix element ρ_{tt} (proportional to the overall fluorescence signal) to $\mathrm{Im}(\rho_{st})$ (proportional to the susceptibility). The susceptibility determines the system's absorption[1] and is associated with the coherent contribution of the fluorescence reduction. In Supplementary Fig. 1(b) we plot ρ_{tt} and $\mathrm{Im}(\rho_{st})$ as a function of Δ_2. This comparison shows that the coherent contri-

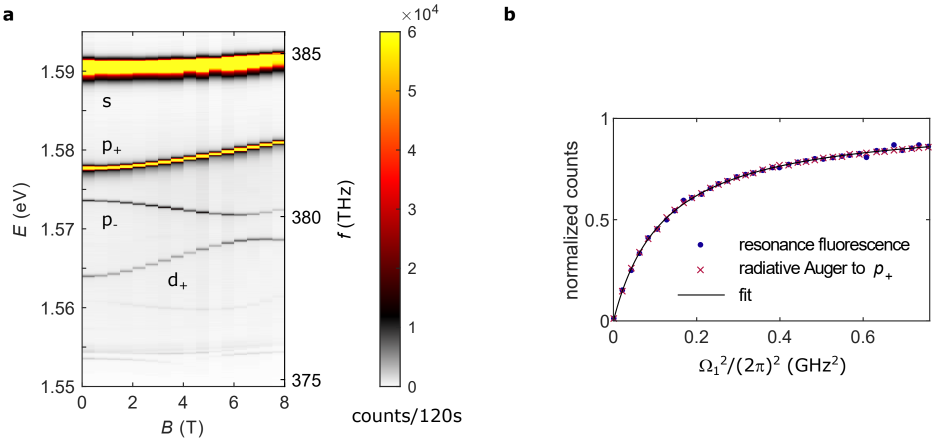

Supplementary Fig. 3. **Magnetic field dependence of the different emission lines.** **(a)** Resonance fluorescence from the fundamental transition and radiative Auger emission as a function of the magnetic field B (data from QD1). The strong magnetic field dispersion of the radiative Auger lines enables direct identification of the final electron states of the Auger electron. **(b)** Normalized fluorescence intensity of the different emission lines as a function of Rabi frequency Ω_1. ω_2 is turned off for this measurement ($\Omega_2 = 0$). The power dependence of the radiative Auger intensity coincides with that of the resonance fluorescence from the fundamental transition and matches the power curve of a two-level system. From a fit to the power curve we determine Ω_1 in our measurements. For fitting, the radiative decay is fixed to a value that we determine from an independent lifetime measurement ($\Gamma_r = 2\pi \times 0.50$ GHz)[5].

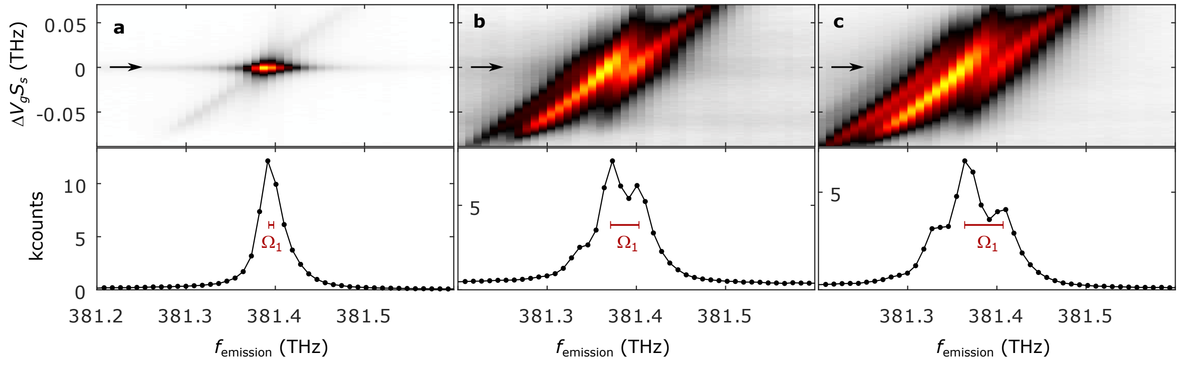

Supplementary Fig. 4. **Radiative Auger emission upon excitation of the fundamental transition.** **(a)** Radiative Auger emission from QD1 (see also Fig. 2(b) of the main text). The emission frequency (x-axis) is plotted as a function of detuning between laser and the $|s\rangle - |t\rangle$ transition (y-axis). In the measurements shown here, the Autler-Townes splitting is not resolved since the Rabi frequency is too small ($\Omega_1 = 2\pi \times 5.5$ GHz). **(b, c)** The same measurements as before performed at higher Rabi frequencies ($\Omega_1 = 2\pi \times 31.9$ GHz, $\Omega_1 = 2\pi \times 43.2$ GHz) where the Autler-Townes splittings are resolved.

bution to the fluorescence reduction (EIT/CPT mechanism) is only part of the overall fluorescence reduction.

SUPPLEMENTARY NOTE 2: MODEL FOR CORRELATION MEASUREMENTS

Time-resolved correlation measurements ($g^{(2)}$-measurements) are used to determine the relaxation time $\tau_p = 1/\Gamma_p$. The corresponding setups are shown in Supplementary Fig. 2(a-c). An auto-correlation of the resonance fluorescence from the fundamental transition and a cross-correlation between emission from the fundamental transition and radiative Auger emission are shown in Supplementary Fig. 2(d). As shown in Supplementary Fig. 2(e), the theoretical model fits well to the data. In these measurements, only a single laser at ω_1 is used. The system is described by Eqs. 1 and 2, with the parameter Ω_2 set to zero. We use the Quantum Toolbox in Python (QuTiP[6]) to compute the steady state density matrix. With the resulting density matrix, we then compute the auto- and the cross-correlation. The auto-correlation is:

$$g^{(2)}(\tau) = \frac{\langle \hat{a}^\dagger(t)\hat{a}^\dagger(t+\tau)\hat{a}(t+\tau)\hat{a}(t)\rangle}{\langle \hat{a}^\dagger(t)\hat{a}(t)\rangle^2}, \tag{3}$$

and the cross-correlation is:

$$g^{(2)}(\tau) = \frac{\langle \hat{a}_A^\dagger(t)\hat{a}^\dagger(t+\tau)\hat{a}(t+\tau)\hat{a}_A(t)\rangle}{\langle \hat{a}^\dagger(t)\hat{a}(t)\rangle\langle \hat{a}_A^\dagger(t)\hat{a}_A(t)\rangle}. \quad (4)$$

In both cases, t is the time and τ is the time delay between two subsequently detected photons. $\hat{a}^\dagger$ describes the creation of a photon via decay into the s-shell (fundamental transition), and $\hat{a}_A^\dagger$ describes the creation of a photon via radiative Auger decay into the excited electron state, $|p\rangle$.

SUPPLEMENTARY NOTE 3: MAGNETIC FIELD DISPERSION OF THE EMISSION

The magnetic field dispersion of the radiative Auger emission is significantly stronger than that of the emission from the fundamental transition (see Supplementary Fig. 3). The reason is the different final state after the optical decay: the electron ground state $|s\rangle$ (s-shell) has a weak magnetic field dispersion and, in contrast, higher shells such as the excited state $|p\rangle$ (p-shell) have a much stronger dependence on the magnetic field. Since the optical emission energy is given by the energy of the trion minus the energy of the final state, the strong magnetic field dispersion is transferred to the radiative Auger lines. The strong dispersion of the radiative Auger emission is an important feature allowing it to be distinguished unambiguously from phonon replicas. For a two-dimensional harmonic confinement potential, the magnetic field dispersions of the different shells form the Fock-Darwin spectrum[7]. The dispersion of the radiative Auger emission is, therefore, typically close to an inverted Fock-Darwin spectrum[3]. A model for the magnetic field dispersion has been developed in Ref. 3.

SUPPLEMENTARY NOTE 4: FURTHER MEASUREMENTS OF AUTLER-TOWNES SPLITTING

In Supplementary Fig. 4 we show additional measurements of the Autler-Townes splitting on QD1. The measurements are performed for different Rabi frequencies Ω_1 and the detuning Δ_1 between laser and fundamental transition is varied. The quantum dot transitions are detuned from the fixed laser by applying a gate voltage, V_g. The detuning from the fundamental transition is $\Delta V \cdot S_s$, where S_s is the Stark-shift of the fundamental transition and ΔV_g the difference in gate voltage. The Rabi frequencies at zero laser detuning are independently determined from a power saturation curve (red bars in Supplementary Fig. 4). They match the measured Autler-Townes splittings in the emission spectra. Furthermore, on detuning the quantum dot resonance from the laser ($\Delta V_g \neq 0$), there is a small probability to excite the trion via the phonon sideband giving rise to a weak "diagonal" emission line. In the case of a red-detuned quantum dot ($\Delta V_g < 0$), the laser has more energy than the quantum dot transition and the additional energy can be transferred to LA-phonons. In the case of a blue-detuned quantum dot, the laser energy is too small and the missing energy can be provided by phonon absorption.

SUPPLEMENTARY REFERENCES

1. Fleischhauer, M., Imamoglu, A. & Marangos, J. P. Electromagnetically induced transparency: Optics in coherent media. *Rev. Mod. Phys.* **77**, 633–673 (2005).
2. Prechtel, J. H. *et al.* Decoupling a hole spin qubit from the nuclear spins. *Nat. Mater.* **15**, 981–986 (2016).
3. Löbl, M. C. *et al.* Radiative Auger process in the single-photon limit. *Nat. Nanotechnol.* **15**, 558–562 (2020).
4. Zibik, E. *et al.* Long lifetimes of quantum-dot intersublevel transitions in the terahertz range. *Nat. Mater.* **8**, 803–807 (2009).
5. Zhai, L. *et al.* Low-noise GaAs quantum dots for quantum photonics. *Nat. Commun.* **11**, 4745 (2020).
6. Johansson, J. R., Nation, P. D. & Nori, F. Qutip 2: A python framework for the dynamics of open quantum systems. *Comput. Phys. Commun.* **184**, 1234–1240 (2013).
7. Kouwenhoven, L. P., Austing, D. G. & Tarucha, S. Few-electron quantum dots. *Rep. Prog. Phys.* **64**, 701–736 (2001).
